# Computed tomography–based pericoronary adipose tissue attenuation in patients undergoing TAVR: a novel method for risk assessment

**DOI:** 10.3389/fcvm.2023.1192093

**Published:** 2023-05-22

**Authors:** Alexandra Steyer, Silvia Mas-Peiro, David M. Leistner, Valentina O. Puntmann, Eike Nagel, Damini Dey, Markus Goeller, Vitali Koch, Christian Booz, Thomas J. Vogl, Simon S. Martin

**Affiliations:** ^1^ Department of Diagnostic and Interventional Radiology, University Hospital Frankfurt, Frankfurt, Germany; ^2^ Institute for Experimental and Translational Cardiovascular Imaging, Goethe University, University Hospital Frankfurt, Frankfurt, Germany; ^3^ Department of Cardiology, University Hospital Frankfurt, Frankfurt, Germany; ^4^ German Centre for Cardiovascular Research (DZHK), Berlin, Germany; ^5^ Cardiopulmonary Institute (CPI), Frankfurt am Main, Germany; ^6^ Biomedical Imaging Research Institute, Cedars-Sinai Medical Center, Los Angeles, CA, United States; ^7^ Department of Cardiology, Friedrich-Alexander-University Hospital Erlangen, Erlangen, Germany

**Keywords:** pericoronary adipose tissue, coronary artery disease, transcatheter aortic valve replacements, computed tomography angiography, risk stratification, major adverse cardiovascular events

## Abstract

**Objectives:**

This study aims to assess the attenuation of pericoronary adipose tissue (PCAT) surrounding the proximal right coronary artery (RCA) in patients with aortic stenosis (AS) and undergoing transcatheter aortic valve replacement (TAVR). RCA PCAT attenuation is a novel computed tomography (CT)–based marker for evaluating coronary inflammation. Coronary artery disease (CAD) in TAVR patients is common and usually evaluated prior to intervention. The most sensible screening method and consequential treatment approach are unclear and remain a matter of ceaseless discussion. Thus, interest remains for safe and low-demand predictive markers to identify patients at risk for adverse outcomes postaortic valve replacement.

**Methods:**

This single-center retrospective study included patients receiving a standard planning CT scan prior to TAVR. Conventional CAD diagnostic tools, such as coronary artery calcium score and significant stenosis via invasive coronary angiography and coronary computed tomography angiography, were determined in addition to RCA PCAT attenuation using semiautomated software. These were assessed for their relationship with major adverse cardiovascular events (MACE) during a 24-month follow-up period.

**Results:**

From a total of 62 patients (mean age: 82 ± 6.7 years), 15 (24.2%) patients experienced an event within the observation period, 10 of which were attributed to cardiovascular death. The mean RCA PCAT attenuation was higher in patients enduring MACE than that in those without an endpoint (−69.8 ± 7.5 vs. −74.6 ± 6.2, *P* = 0.02). Using a predefined cutoff of >−70.5 HU, 20 patients (32.3%) with high RCA PCAT attenuation were identified, nine (45%) of which met the endpoint within 2 years after TAVR. In a multivariate Cox regression model including conventional CAD diagnostic tools, RCA PCAT attenuation prevailed as the only marker with significant association with MACE (*P* = 0.02). After dichotomization of patients into high- and low-RCA PCAT attenuation groups, high attenuation was related to greater risk of MACE (hazard ration: 3.82, *P* = 0.011).

**Conclusion:**

RCA PCAT attenuation appears to have predictive value also in a setting of concomitant AS in patients receiving TAVR. RCA PCAT attenuation was more reliable than conventional CAD diagnostic tools in identifying patients at risk for MACE .

## Introduction

Clinical management of patients undergoing transcatheter aortic valve replacement (TAVR) with concomitant coronary artery disease (CAD) proves challenging in terms of efficient and empirically supported strategies (1). Insufficient data continue to deter the formulation of robust recommendations for the wider integration of computed tomography (CT)-based CAD evaluation preceding intervention (2). Consequently, immoderate diagnostic approaches prevail and await further scrutiny as TAVR-receiving cohorts evolve and the ambiguity surrounding concurrent CAD grows (3). The possibilities of CT-based CAD evaluating tools have expanded in the last few years with positive signals, particularly for a rule-out approach during conventional TAVR workup (4, 5). Identifying patients at risk for adverse cardiovascular events from this prevalent comorbidity and its interactivity has proven to be cumbersome, underlining the apparency of a missing link in current stratification techniques (6, 7).

Pericoronary adipose tissue (PCAT) attenuation is one of the several novel CT-based tools for assessing CAD with the added advantage of insight into the activity of atherosclerotic inflammation and consecutive plaque instability (8). The CT-based measurement of adipose tissue attenuation around a defined perivascular space, particularly of the proximal right coronary artery (RCA), was shown to be reflective of the proinflammatory interplay between cytokine and adipocyte differentiation with a subsequent deviation of tissue density (9, 10). To which extent aortic stenosis (AS) interferes with regular pathophysiological processes of CAD, particularly in terms of coronary inflammation remains largely unexplored (11). Unlike the well-established coronary artery calcium score (CACS) and stenosis quantification, the utility of PCAT is yet to be examined in patients undergoing TAVR (4, 12, 13). Recently, a substudy of the large multicenter SCOT-HEART trial illustrated RCA PCAT as an independent predictor of myocardial infarction (MI) in patients with suspected CAD within a 5-year follow-up period (14). This study will investigate whether this tool is reliable in patients with bystanding AS and could function as a marker for better placement of patients at risk for adverse cardiovascular outcomes. We also aim to examine the relationship between PCAT and other conventional CAD diagnostic tools, such as the CACS, coronary computed tomography angiography (CCTA)–based stenosis quantification, and invasive coronary angiography (ICA).

## Methods

### Study design

**Figure 1:**
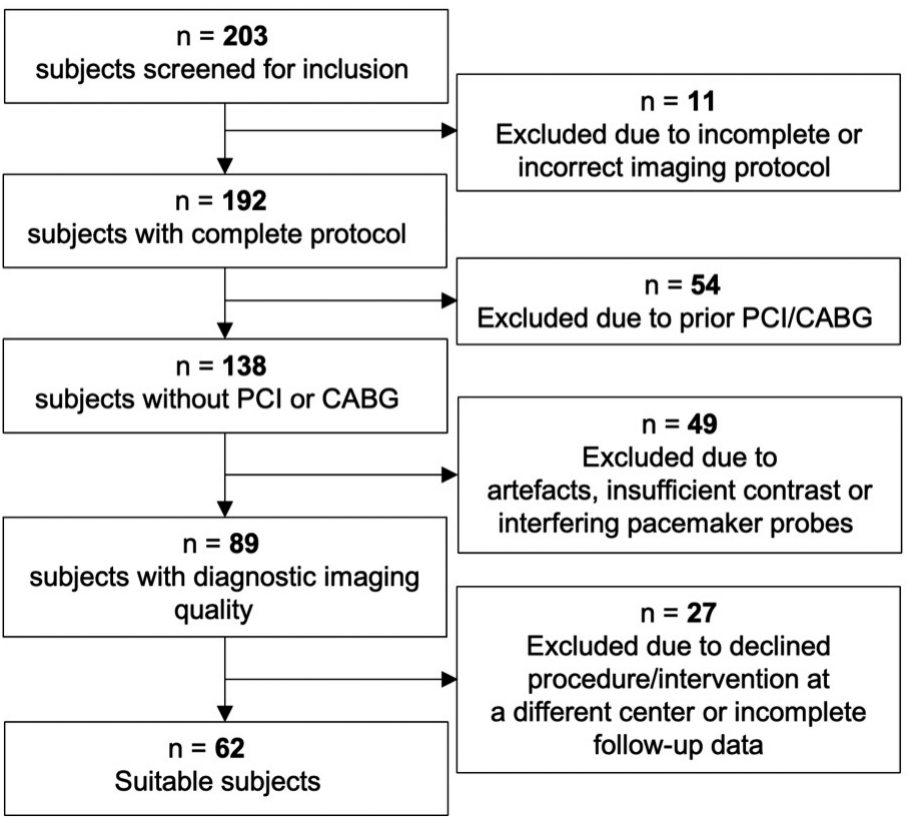
Between 2019 and 2020, 203 patients receiving TAVR planning were evaluated for enrollment in the study. A total of 18 patients were excluded due to declined procedure or final intervention received at a different center, a further 9 patients had incomplete follow-up data. PCI, percutaneous coronary intervention; CABG, coronary artery bypass grafting.

This single-center, retrospective study was conducted on patients undergoing TAVR between January 2019 and January 2020 at the University Hospital Frankfurt, Germany. Patients receiving preprocedural planning during 2019 were visually screened and assessed for study inclusion and observed throughout a 24-month follow-up period. The imaging data were acquired in the clinical routine. Inclusion criteria consisted of a complete, standardized planning protocol, sufficient imaging quality, and an absence of foregoing invasive CAD treatment in the form of percutaneous coronary intervention (PCI) or coronary artery bypass graft surgery (CABG). For consistency purposes, only examinations conducted with a tube voltage of 100, 110, and 120 kV were considered for inclusion (15). Furthermore, ICA and CCTA had to be performed within 6 weeks of TAVR intervention. Patients with foreign materials, such as pacemaker probes that lead to debilitating artifacts, were excluded. A summary of the patient selection process can be found in Figure 1. The primary endpoint was defined as a composite of cardiovascular death, stroke, and myocardial infarction. The study protocol was approved by the institutional ethics committee, and patient-related information was handled according to the European General Data Protection Regulation (EU-GDPR). All patients supplied written informed consent for the use of data for general research purposes within the institute.

### Imaging protocol

All examinations were conducted with a single third-generation 2 × 192 slice dual-source CT scanner (SOMATOM Force, Siemens Healthineers, Erlangen, Germany). A conventional calcium scan with a 120 kV automatic tube current modulation (CARE Dose 4D, Siemens Healthineers) was performed first. Next, 90 mL of contrasting agent (400 mg/mL iomeprol; Iomeron, Bracco, Milan, Italy) at a 5 mL/s injection rate was applied in succession with optimal image acquisition timing via bolus tracking. A prospective high-pitch spiral electrocardiographic (ECG) gating technique, following a manual review of test bolus peak registration, was used. The high concomitance of atrial fibrillation and restrictive premedication use repeatedly necessitated retrospective ECG triggering with full dose application between the early systole and early diastole. The tube voltage was adapted automatically according to patient-specific features (CARE kV, Siemens Healthineers), and a collimation of 0.6 mm was used. The institute's TAVR planning protocol was adapted from the Society of Cardiovascular Computed Tomography (SCCT) consensus paper guidelines (16). All imaging data were exported to the institute's picture archiving and communication system server before being analyzed at a designated workstation.

### CAD diagnostic tools

**Figure 2:**
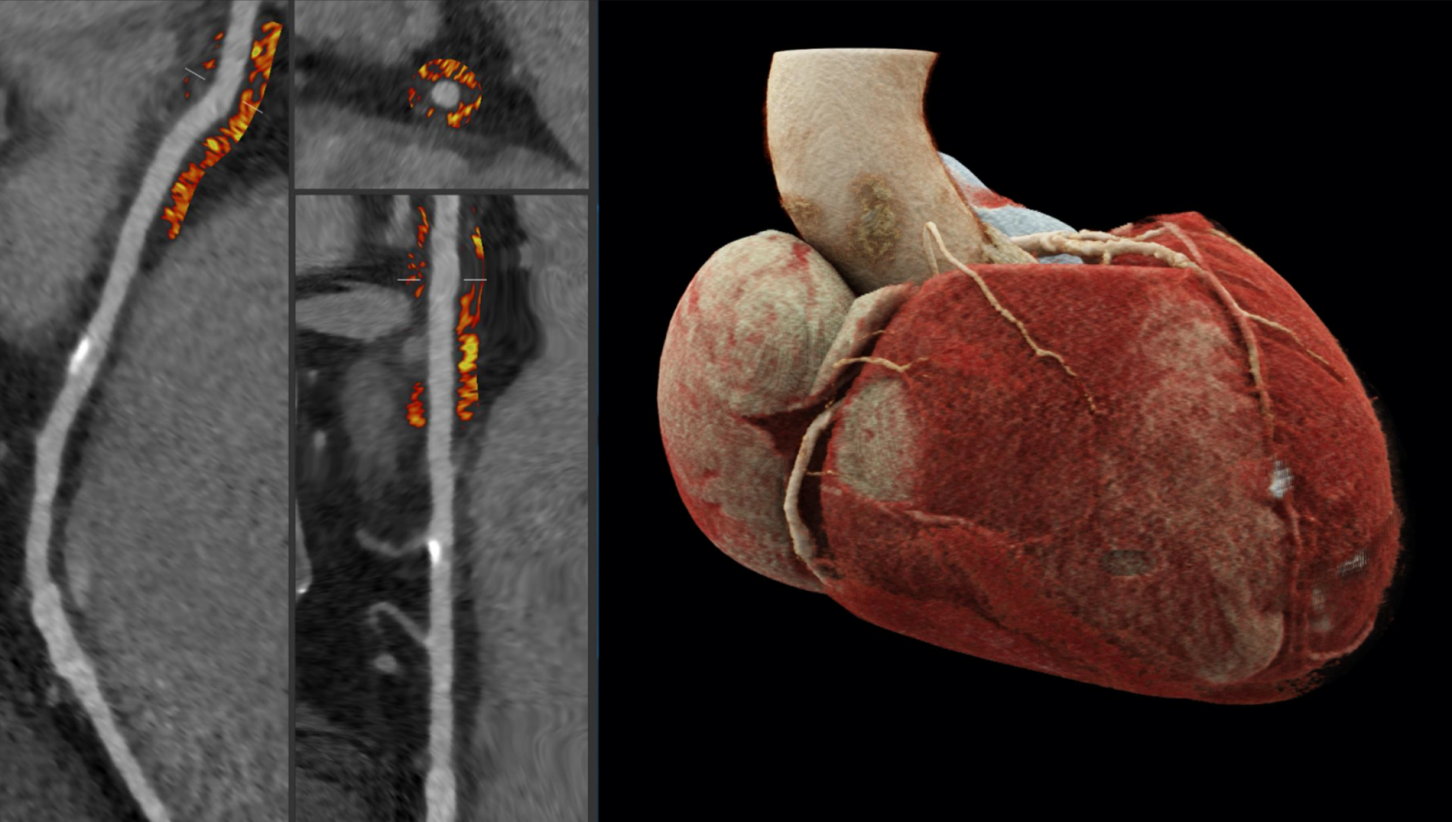
To the left side, examples of cross-sectional and straightened reconstructions with color mapping expressive of PCAT density. The average attenuation of the perivascular adipose tissue surrounding the outer vessel wall within a voxel frame of -190 to -30HU proximal to the RCA is determined; an exemplary calcified plaque can be found a few centimeters distally to the region of interest. The image to the right demonstrates a three-dimensional reconstruction of a heart via cinematic rendering, the RCA can be seen emerging from the aorta and descending the atrioventricular groove, the perivascular adipose tissue of the first 10-50mm of the vessel is considered for PCAT computation.

PCAT analyses were performed using semiautomated software (Autoplaque, version 2.5, Cedars-Sinai Medical Center, CA, USA). The perivascular region equal to the width of the average RCA diameter within the first 10–50 mm of the vessel was assessed via the identification of control points in cross-sectional and multiplanar reformations. The average attenuation of voxels within a frame of −190 to −30 HU defines the RCA PCAT attenuation. The analysis was conducted as described previously (8, 14, 17). An example is shown in Figure 2. The CCTA-based stenosis quantification was performed using dedicated semiautomated software (syngo.CT Coronary Analysis, Siemens Healthineers). Suggested vessel centerlines from curved planar reconstructions were manually edited as were the luminal contours using cross-sectional segments. The stenosis grade was computed for lesions in vessels with a diameter >1.5 mm and considered significant when ≥50% (≥CAD-RADS 3) (18). CACS was determined using automated software (syngo.CT CaScoring, Siemens Healthineers). All examiners were blinded to all patient-related clinical data while performing the postimaging analyses. Lastly, ICA was performed before TAVR intervention and was also considered relevant in the presence of luminal stenosis ≥50%.

### Statistical methods

Statistical analyses were performed using SPSS version 29.0 (IBM SPSS Statistics, Chicago, IL, USA). Continuous variables were assessed for normality using the Shapiro–Wilk test. In cases of a normal distribution, the variables were reported with mean ± standard deviation (SD), and if not, normally distributed median and interquartile ranges (IQR) were used. For hypothesis testing, the Student's *t*-test and Mann–Whitney *U*-test were used. Categorical variables were presented as frequencies (percentages), and a Pearson's chi-square test was applied for comparison between variables. The correlation between two continuous variables was assessed using Pearson's correlation coefficient. Outcome-related data were evaluated using a Cox proportional hazard model via both univariate and multivariate analyses and displayed with corresponding hazard ratios, confidence intervals, and significance levels. The univariable Cox proportional hazard regression was used to identify clinical variables associated with the primary outcome. A multivariate model was constructed using conventional CAD evaluation tools, such as CACS, significant stenosis in ICA and CCTA, and RCA PCAT attenuation, where all covariates were forced into the model (19). A graphic representation of a Cox proportional hazard analysis for RCA PCAT using a previously described cutoff threshold of −70.5 HU (14) was displayed in the form of a cumulative incidence plot. A two-sided *P*-value of <0.05 was considered statistically significant.

## Results

### Population

This Caucasian TAVR cohort consisted of 45% females with an overall mean age of 82 ± 6.7 years. Arterial hypertension and atrial fibrillation were the two most common comorbidities in 48/62 (77.4%) and 28/62 (45.2%) patients, respectively. All baseline characteristics stratified according to RCA PCAT CT attenuation are summarized in Table 1. This was the original sentence, we ask to keep it this way as we believe it sounds less wordy: BMI, EuroscoreII, troponin T, and creatinine were higher in patients with high RCA PCAT attenuation, the remaining clinical and patient-specific features were comparable across both groups.

**Table 1:** Baseline demographics and clinical features stratified according to RCA PCAT attenuation.

Patient characteristics at study baseline	RCA PCAT attenuation >−70.5 (*n* = 20)	RCA PCAT attenuation ≤−70.5 (*n* = 42)	*P*-value
Age, years	83.9 ± 6.0	81.4 ± 6.9	0.23
Female (%)	7 (35.0)	20 (47.6)	0.35
BMI, kg/m²	27.7 ± 5.3	31.5 ± 7.3	0.005
**Medical history, *n* (%)**			
Hypertension	16 (80.0)	32 (76.2)	0.74
Atrial fibrillation	9 (45.0)	19 (45.2)	0.99
Stroke or TIA	2 (10.0)	3 (7.1)	0.72
Diabetes mellitus	9 (45.0)	16 (38.1)	0.60
Peripheral vascular disease	2 (10.0)	5 (11.9	0.83
**Cardiac assessment**			
NYHA class			
I	0	1 (2.4)	0.49
II	4 (20.0)	10 (23.8)	0.74
III	12 (60.0)	30 (71.4)	0.37
IV	4 (20.0)	1 (2.4)	0.02
EuroSCORE II	5.1 (3.0–17.6)	2.4 (1.6–3.8)	<0.001
Previously diagnosed CAD (%)	8 (40.0)	12 (28.6)	0.39
**Clinical features**			
CRP, mg/L	0.5 (0.06–10.7)	0.3 (0.04–2.9)	0.474
Leukocytes, /µL	7.5 (5.9–9.0)	7.0 (5.0–7.7)	0.537
Troponin T, pg/mL	29.0 (15.5–54.5)	18.2 (14.7–25)	0.011
Creatinine, mg/dL	1.8 ± 1.4	1.0 ± 0.4	0.001
Receiving statin therapy, *n* (%)	16 (80.0)	27 (64.3)	0.21

The results are reported as *n* (%), mean ± SD, or median (range) as appropriate and rounded to the first decimal place. AU, arbitrary units; BMI, body mass index; stroke or TIA, transitory ischemic attack; NYHA class, New York Heart Association class; CAD, coronary artery disease; CRP, C-reactive protein.

### CAD assessment

Significant stenosis was found in 22 (35.5%) patients via ICA, of which 13 patients had single-vessel disease, five had a two-vessel disease, and four had a three-vessel disease. CCTA distinguished 40 (64.5%) patients with significant stenosis, of which 19 patients had stenoses ≥70%. In 36 (58.1%) patients, ICA and CCTA findings were coherent. CAD was newly diagnosed in eight patients during TAVR planning using ICA as the reference standard. PCI was performed in four patients preceding the TAVR intervention. The overall median CACS was 277.4 (79.6–836.1). CACS was high (>400 HU) in 17/22 patients with significant stenosis in ICA and was associated with stenosis both in ICA and CCTA (*P* = 0.011 and *P* = 0.043). The extent of CACS was insignificantly associated with the occurrence of major adverse cardiovascular events (MACE). Similarly, the presence of significant stenosis in ICA or CCTA also showed no relevant association with MACE; see Table 2 for a summary of all CAD markers in relation to the primary outcome.

**Table 2:** CAD assessment in accordance with outcome grouping.

CAD assessment tool	Event, *n* = 15	No event, *n* = 47	*P*-value
Significant stenosis in ICA, (%)	6 (40.0)	16 (34.0)	0.68
Significant stenosis in CCTA, (%)	10 (66.7)	30 (63.8)	0.84
CACS, AU	539.7 (149.4–1,669.3)	192.7 (56.0–792.0)	0.10
RCA PCAT attenuation, HU	−69.8 ± 7.5	−74.6 ± 6.2	0.02

The results are reported as *n* (%), mean ± SD, or median (IQR) as appropriate and rounded to the first decimal place. AU, Agatston units; HU, Hounsfield units; ICA, invasive coronary angiography; CCTA, coronary CT angiography; CACS, coronary artery calcium score.

### RCA PCAT attenuation

Overall RCA PCAT attenuation was normally distributed around a mean of −73.41 ± 6.86 HU. Patients with relevant CAD in preprocedural ICA had a trend for higher average RCA PCAT attenuation than that those with no visible CAD (−71.59 HU vs. −74.72 HU, *P* = .076). As for CCTA, the difference in mean RCA PCAT attenuation was negligible (−73.14 HU vs. −74.07 HU, *P* = 0.632). The correlation between CACS and RCA PCAT was insignificant (*r* = −.005, *P* = 0.97), as was the correlation to systemic inflammation markers, i.e., C-reactive protein (CRP) and leukocyte count (*r* = 0.191, *P* = 0.137 and *r* = 0.63 *P* = 0.626). A moderate positive correlation was demonstrated for the EuroSCORE II and serum troponin T (*r* = 0.42, *P* = <0.001 and *r* = 0.30, *P *= 0.016). Gender or ongoing statin therapy did not influence RCA PCAT attenuation. Using a previously described predictive threshold of −70.5 HU, a total of 20 (32.3%) patients with potentially elevated risk for adverse outcomes were distinguished. We believe this is less wordy: Within this group 11 (55%) patients had relevant CAD in ICA, MACE was encountered by 9 (45%) patients and was significantly associated with a high RCA PCAT attenuation (*P* = .008). Similarly, all-cause death was associated with RCA PCAT attenuation above this threshold (*P* = 0.002).

### Outcome

**Figure 3:**
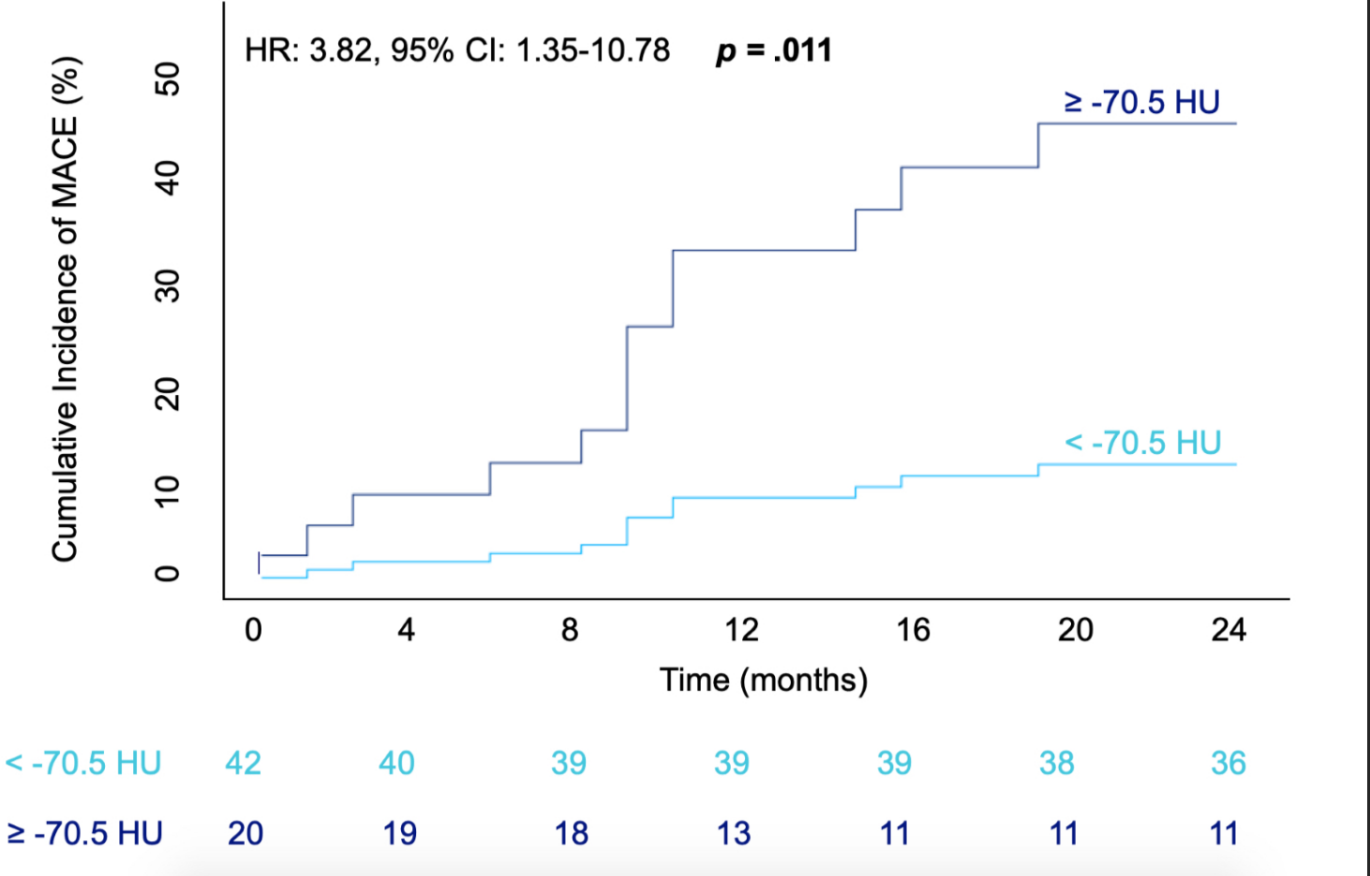
Cumulative incidence of MACE within 24-months after TAVR intervention in accordance with RCA PCAT Attenuation. Graphical representation of a Cox proportional hazards model; patients with attenuation ≥-70.5HU depicted via the dark blue line, those with attenuation values of <-70.5HU in light blue. The number of patients with MACE-free survival at a four monthly interval are displayed underneath.  The hazard ratio, confidence interval and corresponding p value are shown in the top left corner of the graph. MACE = major adverse cardiovascular event; HR = Hazard ratio; CI = confidence interval; HU= Houndsfield units.

During the 24-month follow-up, 15 (24.2%) patients met the endpoint, the most common cause being cardiovascular death in a total of 10 patients. Cardiovascular death was attributed to heart failure in five patients (50%), acute cardiac death in two patients and in three patients no further specification was available. Non-fatal stroke occurred in five patients; only one patient experienced myocardial infarction. Two-thirds of all events occurred within the first 12 months (Figure 3). Univariable analyses identified CRP and troponin T as predictive clinical characteristics for MACE (Table 3). Furthermore, the EuroSCORE II and male sex also functioned as predictors of the outcome.

**Table 3:** Univariate associations of clinical and patient features with MACE.

Clinical feature	HR (95% CI)	*P-*value
Age, years	1.01 (0.93–1.10)	0.76
Male	5.76 (1.30–25.55)	0.02
BMI	0.94 (0.85–1.03)	0.19
Arterial hypertension	0.81 (0.26–2.55)	0.72
Diabetes mellitus	1.84 (0.67–5.08)	0.24
Known CAD	0.48 (0.14–1.70)	0.25
EuroSCORE II	1.00 (1.00–1.01)	0.07
CRP	1.35 (1.11–1.66)	0.003
Leukocytes	1.00 (0.98–1.02)	0.71
Troponin T	1.03 (1.01–1.04)	<0.001
Serum creatinine	1.04 (0.9–1.16)	0.52

Analyses performed via Cox proportional hazard regression, *n* = 62. CI, confidence interval; HR, hazard ratio; BMI, body mass index; CAD, coronary artery disease; CRP, C-reactive protein.

**Figure 4:**
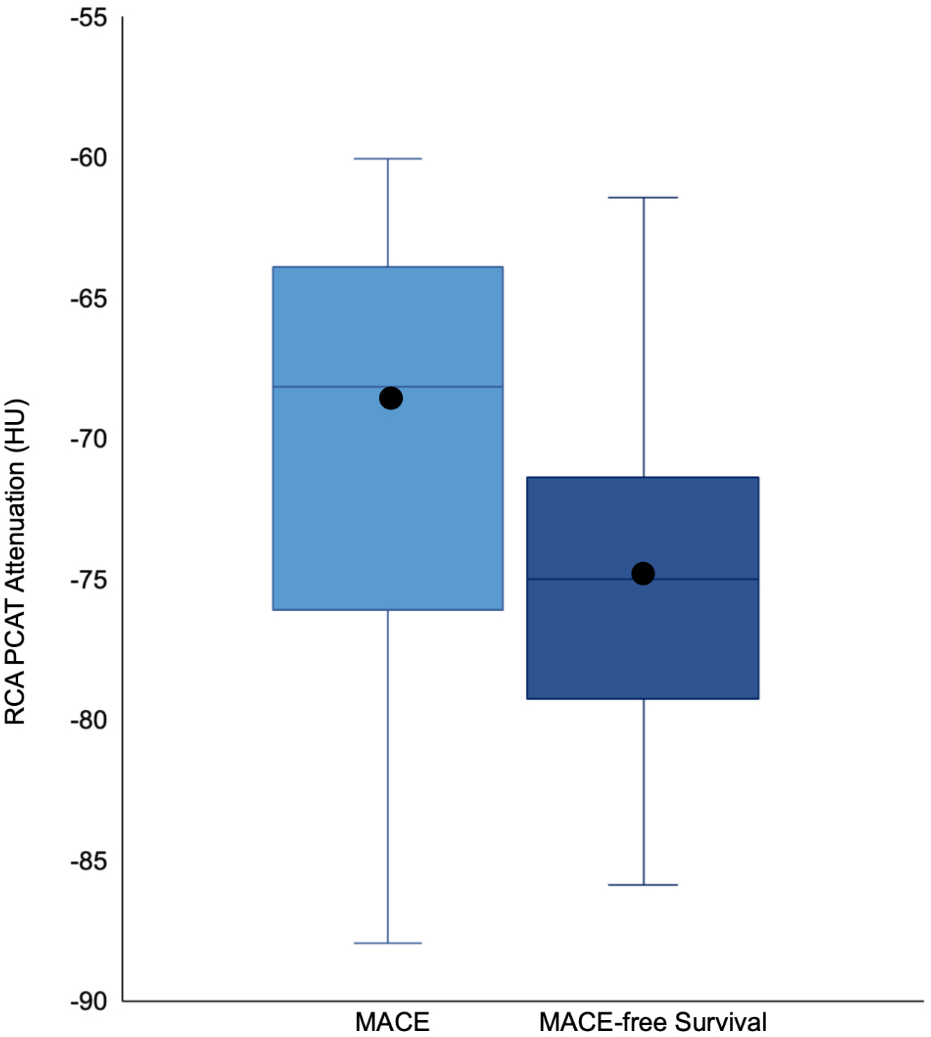
Boxplots illustrating the difference in median and distribution of RCA PCAT Attenuation in patients with and without MACE. The box represents the interquartile range, the whiskers indicate the range. The medians are represented by the horizontal black lines, the means via black circles. RCA PCAT attenuation was higher in patients with an event (-69.8 ±7.5) compared to patients without MACE (74.6 ± 6.2). RCA PCAT = right coronary artery pericoronary adipose tissue. All other abbreviations as in Figure 3.

Patients who encountered MACE had a higher RCA PCAT attenuation compared to that of those that did not meet the primary outcome (−69.8 ± 7.5 vs. −74.6 ± 6.2, *P* = 0.02) (Figure 4 and Table 2). Correspondingly, patients experiencing cardiovascular death had significantly higher RCA PCAT attenuation (−67.2 ± 5.5 vs. −74.6 ± 6.5, *P* = 0.001). As for the isolated endpoint stroke no deviation of the mean RCA PCAT could be shown. Neither significant stenosis distinguished via ICA and CCTA nor CACS was associated with MACE (Table 2). In multivariable analyses including all examined CAD tools, RCA PCAT attenuation prevailed as the only significant predictor of MACE within 24-month post-TAVR [hazard ratio (HR): 1.11, *P* = 0.015] (Table 4). The area under the curve for RCA PCAT attenuation for the prediction of MACE was 0.705 (IQR: 0.54–0.87, *P* = 0.018) in receiver operating characteristic (ROC) analyses. Following the dichotomization of patients into high- and low-RCA PCAT attenuation groups, a decrease in event-free survival for patients with RCA PCAT ≥−70.5 HU attenuation was shown in Cox proportional hazard regression analysis (HR: 3.82, 95% CI 1.35–10.78, *P* = 0.011) (Figure 3).

**Table 4:** Multivariable associations of CAD assessing tools with MACE.

	Univariable analyses		Multivariable analyses	
CAD assessment tool	HR (95% CI)	*P-*value	HR (95% CI)	*P-*value
Significant stenosis in ICA, (%)	1.16 (0.41–3.26)	0.780	0.70 (0.22–2.28)	0.557
Significant stenosis in CCTA, (%)	1.10 (0.38–3.221)	0.857	0.96 (0.30–3.10)	0.942
CACS, AU	1.00 (1.00–1.00)	0.318	1.00 (1.00–1.01)	0.200
RCA PCAT attenuation, HU	1.09 (1.01–1.18)	0.022	1.11 (1.02–1.20)	0.015

The model was constructed via Cox proportional hazard regression; conventional CAD evaluating tools as well as the novel RCA PCAT attenuation were incorporated. *n* = 62. HR, hazard ratio; CI, confidence interval; AU, Agatston units; HU, Hounsfield units; ICA, invasive coronary angiography; CCTA, coronary CT angiography; CACS, coronary artery calcium score.

## Discussion

In this retrospective study, which is, to our knowledge, the first to examine RCA PCAT attenuation in patients undergoing TAVR, an association between higher attenuation values and the composite endpoint MACE within 2 years after intervention could be shown. Compared to conventional CAD diagnostic tools, RCA PCAT attenuation was the only marker with relevant predictive value for an adverse cardiovascular outcome post-TAVR.

Classically utilized diagnostic tools, such as stenosis degree and CACS, were not associated with the outcome, this was unlike prior studies evaluating RCA PCAT attenuation in a general patient population with suspected CAD (14, 20). The modest specificity of significant stenosis determined via CCTA in this setting is known and stems in part from the analogous yet oversensitive use of invasive stenosis grading for this modality (21, 22). More essentially, however, stenosis degree whether determined via ICA or CCTA provides a snapshot of the present obstruction across a lesion with little information about dynamic inflammatory processes or hemodynamic consequences (23). Furthermore, most published works evaluating RCA PCAT attenuation with, and in comparison to, general CAD tools examine patients with present symptoms of CAD and, thus, the greater likelihood of active disease than is the case for AS patients not infrequently receiving first diagnosis during TAVR planning.

We used a previously determined threshold as an orientation for the extrapolated use in this comorbid cohort (14). The cutoff of −70.5 HU defined using the same software is near the −70.1 HU from the validating CRISP-CT study (10). Only examinations conducted with tube voltages of 100-120 kV were included, as deviation from these results in varying attenuation of perivascular adipose tissue, with no existing verified correction factors as of yet (10, 15). The average RCA PCAT attenuation in this TAVR cohort was slightly higher than reported in previous non-AS cohorts (10, 14, 20, 24). Whether higher pericoronary fat density is a consequence or collateral effect from severe AS is largely unexplored and non-deductible from this work (25). Concomitant AS with inherent inflammatory properties resembling that of atherosclerosis (26, 27) plausibly interacts with atherogenesis and its effects on perivascular adipose tissue. Resultantly, implementing a cutoff value higher than previously defined in the foregoing studies with sole CAD patients may be rational. Serious deduction of a potentially deviating RCA PCAT threshold in AS patients would, however, necessitate a larger population than that examined in this study. Nonetheless, a preliminary ROC analysis in the present study demonstrated robust discrimination using the abovementioned threshold. Another noteworthy feature was the notably older study population compared to that of preceding studies (10, 14, 20). Aging influences the composition of pericoronary fat with a potential effect of the higher measured attenuation values in these findings (28). Tzolos et al. found no difference in RCA PCAT attenuation after adjustment for age, wherein the proportion of patients above the age of 70 years was however small. Prediction of MACE using RCA PCAT attenuation was independent of CACS and stenosis severity whether determined via ICA or CCTA. A multivariate proportional hazard model was constructed incorporating these conventional tools to adjust for the impact of each instrument and assess the stability of their effect. These findings were consistent with those from two large cornerstone studies examining this method in patients with suspected CAD (10, 14). Tzolos et al. reported a significant association between obstructive CAD and risk for MI. This was influenced, however, by the presence of low-attenuation plaque burden and RCA PCAT attenuation. In addition, RCA PCAT attenuation was shown to correlate with CCTA stenosis defined as >70%, a relationship that could not be supported by our findings. Similarly, Antonopoulos et al. illustrating the biological properties of perivascular adipose tissue and the interrelation to CT detectable inflammatory changes showed deviated PCAT attenuation disregarding obstructive or non-obstructive disease status (8). In terms of CACS, no association with RCA PCAT attenuation could be shown in this present study, as congruent with previous findings (8, 10). CACS tends to progress with a slight tendency of resorption after deposition, hence allowing for restricted assessment of change in the activity of inflammation (8, 29). Correspondingly, luminal stenosis develops on the basis of coronary inflammation; It is, however, the unstable nature of the plaque that facilitates acute ischemia or incremental worsening of a chronic coronary syndrome. This is where RCA PCAT attenuation might prove to be more responsive to changes in the level of inflammation (17, 24) or early atherosclerosis with undeveloped plaque formation (8). Systemic inflammation markers, CRP, and white blood count were not significantly altered in patients with higher RCA PCAT attenuation, in fitting with previous findings supporting the notion of a primarily local atherogenic phenomenon (14, 23). Elevated CRP and white blood count were, however, also associated with MACE in this present study (24).

RCA PCAT attenuation, initially developed to evaluate risk arising particularly from CAD in the form of acute coronary syndrome and MI, appeared to have predictive value for general causes of cardiovascular death (10). Our results corresponded with those from previous groups examining RCA PCAT attenuation in relation to a composite of cardiac death causes (10, 20) rather than MI exclusively (14). These findings supported the perception that TAVR patients have an inherent cardiovascular risk that is debatably potentiated by the presence of CAD (1). Only one MI was recorded within the follow-up duration ensuing in insignificant associations of this individualized endpoint; this was, however, a comparable post-TAVR event rate to that reported by the large multicenter PARTNER 3 trial (30). The event rate, particularly for MI postintervention, may be relatively low, the prognosis nonetheless meager (30). Furthermore, it remains unexplored whether this event rate increases several years postintervention, with little outcome-related data past the 5-year postintervention mark (31).

The meaning of CAD in the long term may grow in relevance as increasing numbers of younger, lower-risk patients receive TAVR. CAD progression in a setting of concomitant AS is likely to assume different pathophysiological mechanisms and develop unlike solitary disease; hence, it seems reasonable to extend observation periods and anticipate a different course of disease in these patients several years after intervention. A potential overshadowing effect from comorbidity and frailty on clinical outcomes and the prediction of them rather than being explicitly CAD-related should, however, also be considered. This holds true particularly for RCA PCAT attenuation which appears to be indicative of general adverse cardiac outcomes. With all things considered, RCA PCAT attenuation is a simple, low-demand risk assessment tool enabling objective identification of patients with greater hazard for an adverse event after TAVR intervention.

### Study limitations

This was a relatively small single-center study with a sufficient overall event rate, however, with only one myocardial infarction; this should be considered when interpreting outcome-related analyses regarding strict CAD affiliation. Interpretation of the multivariate analyses should be done cautiously. We tried to be restrictive with the inclusion of only conventionally utilized CAD diagnostic tools; nonetheless, this was a rather small data set and thereupon few events. Furthermore, this is a novel tool with little existing clinical data and more groundwork to be covered regarding the pathophysiologic interpretation, particularly in the case of concomitant AS. For some analyses, dichotomization of patients into high- and low-RCA PCAT attenuation groups was conducted using a threshold determined within a non-AS cohort.

## Conclusion

In summary, this was a preliminary study that examined the prospects of RCA PCAT attenuation application in AS cohorts undergoing TAVR. These first analyses suggested the viability of this method, previously established in general CAD populations, also in this setting. The findings from this work were compatible with those from previous larger, validating studies, suggesting the legitimacy of this novel image-based biomarker in cases of bystanding AS.

## Data Availability

The original contributions presented in the study are included in the article; further inquiries can be directed to the corresponding author.
